# AHNAK2 Is Associated with Poor Prognosis and Cell Migration in Lung Adenocarcinoma

**DOI:** 10.1155/2020/8571932

**Published:** 2020-08-20

**Authors:** Shusen Zhang, Yuanyuan Lu, Lei Qi, Hongyan Wang, Zhihua Wang, Zhigang Cai

**Affiliations:** ^1^Department of Respiratory and Critical Care Medicine, The Second Hospital of Hebei Medical University, Shijiazhuang, Hebei, China; ^2^Department of Respiratory and Critical Care Medicine, Affiliated Xing Tai People Hospital of Hebei Medical University, Xingtai, Hebei, China; ^3^Department of Anesthesiology, Affiliated Xing Tai People Hospital of Hebei Medical University, Xingtai, Hebei, China; ^4^Department of Pathology, Affiliated Xing Tai People Hospital of Hebei Medical University, Xingtai, Hebei, China; ^5^Department of Thoracic Surgery, The Fourth Hospital of Hebei Medical University, Shijiazhuang, Hebei, China

## Abstract

**Background:**

Lung adenocarcinoma (LUAD), as the main subtype of lung cancer, is one of the common causes of cancer-related deaths worldwide. The AHNAK family is correlated with cell structure and migration, cardiac calcium channel signaling, and tumor metastasis. Previous studies showed AHNAK2 could promote tumor progression and cell migration in melanoma and renal clear cell carcinoma. However, the role of AHNAK2 in LUAD remains unknown.

**Methods:**

We examined the levels of AHNAK2 in pathological specimens and the database of Clinical Proteomic Tumor Analysis Consortium-Lung adenocarcinoma (CPTAC-LUAD), The Cancer Genome Atlas-Lung Adenocarcinoma (TCGA-LUAD), Gene Expression Omnibus dataset (GSE72094, GSE26939), and The Genotype-Tissue Expression (GTEx) of lung tissue samples. Univariate Cox regression, multivariate Cox regression, and Kaplan–Meier survival analysis were performed to reveal the relationship between AHNAK2 and prognosis. A nomogram was constructed to predict 2- or 3-year overall survival and validated via calibration curves, receiver operating characteristic (ROC) analysis, and decision curve analysis (DCA). Furthermore, Gene Ontology (GO) analysis and Kyoto Encyclopedia of Genes and Genomes (KEGG) analysis were used to explore the functional role of AHNAK2 in lung adenocarcinoma. Finally, by transfecting siRNA, we examined the regulatory effect of AHNAK2 on cell migration.

**Results:**

The expression of AHNAK2 was upregulated in tumor samples and correlated with poor prognosis in LUAD patients. Nomogram with AHNAK2 and clinical parameters showed a good prediction in overall survival (OS), especially the 2-year OS. In addition, functional analyses and wound healing assay suggested that AHNAK2 might be involved in the regulation of migration in LUAD.

**Conclusion:**

In summary, our study showed that AHNAK2 might be a novel biomarker in LUAD and revealed the potential mechanism of AHNAK2 in LUAD progression which could provide new insights for target therapy.

## 1. Introduction

Lung cancer is one of the most common cancers that seriously threaten human health [1, 2]. One authoritative statistical survey on lung cancer showed that there were 2.09 million new patients and more than 1.76 million deaths worldwide in 2018, ranking first in both the incidence and mortality [3]. Lung adenocarcinoma (ADC) is the main subtype of lung cancer [4, 5]. In recent years, the diagnosis and treatment methods have been continuously improved, for example, the application of targeted therapy has improved many patients' quality of life [6]. However, the overall survival rate of lung adenocarcinoma is not significantly upgraded [7]. Therefore, it is imperative to conduct researches on the mechanism and identify powerful biomarkers of LUAD.

AHNAK2 (AHNAK nucleoprotein 2), also known as C14orf78, is a member of the AHNAK family, which also includes its homologous gene AHNAK [8]. AHNAK2 was originally found in mouse heart tissue extract and encode a giant protein of more than 600 KDa and included three domains, i.e., the N-terminal PDZ domain, the central repeating units (CRUs) region, and the C-terminal domain [9, 10]. AHNAK plays a key role in regulating blood-brain barrier formation, cell structure and migration, cardiac calcium channel signaling, and tumor metastasis, while there is a relative lack of functional research on AHNAK2, a homologue of AHNAK [11, 12]. Upregulated ANHAK2 activates the PI3K/AKT signaling pathway and promotes melanoma cell metastasis [13]. Wang et al. reported that depletion of AHNAK2 inhibited lipid synthesis and further inhibited the metabolism of renal clear cell carcinoma cells. Moreover, in hypoxic environment, HIF-1*α* (hypoxia inducible factor-1*α*) induces the upregulation of AHNAK2 levels and promotes the progression of renal clear cell carcinoma by promoting epithelial-mesenchymal transition (EMT) [14]. Recent studies revealed that ANHAK2 could act as a biomarker for various tumors, such as thyroid cancer, pancreatic ductal cancer, bladder cancer, and gastric cancer [15–18]. However, the specific role of AHNAK2 in lung adenocarcinoma remains unknown.

In this investigation, we detected AHNAK2 levels in pathological specimens and databases from Clinical Proteomic Tumor Analysis Consortium (CPTAC), The Cancer Genome Atlas (TCGA), Gene Expression Omnibus (GEO), and The Genotype-Tissue Expression (GTEx). Moreover, we found that overexpression of AHNAK2 was significantly associated with poor prognosis in lung adenocarcinoma. Furthermore, nomogram was built to predict 2- or 3-year overall survival for LUAD patients. In addition, using datasets from Broad Institute Cancer Cell Line Encyclopedia (CCLE) lung adenocarcinoma cell lines and TCGA-LUAD, we explored the relation functions and pathways with AHNAK2 in ADC. Finally, we found that knockdown of AHNAK2 restrained ADC cell migration. Our findings showed that AHNAK2 could be a novel prognostic marker and therapeutic target of lung adenocarcinoma.

## 2. Materials and Methods

### 2.1. Tissue Array and Immunohistochemistry

The tissue microarray of 80 LUAD tumor and adjacent nontumor samples were obtained from SuperBiotek (Shanghai, China). Immunohistochemistry was performed for AHNAK2 on the specimens by anti-AHNAK2 antibody (HPA004145; Sigma-Aldrich, St. Louis, MO, USA, diluted 1 : 500). The intensity of staining was scored as 0 (negative), 1 (weak), 2 (moderate), or 3 (strong) [19]. The extent of staining was scored according to the percentage of positive tumor cells: 0 (none), 1 (1–10%), 2 (11–50%), 3 (51–75%), and 4 (>75%). The multiply of the two scores ranged 0-12, and 0-4 were considered as low expression, while ≥6 was considered as high expression. The paired difference plot was used to compare the expression of AHNAK2 in tumor to that in adjacent nontumor tissues.

### 2.2. Data Profile Collection

The protein expression of AHNAK2 was obtained from CPTAC-LUAD proteome which contained 102 normal samples and 109 tumor samples (https://cptac-data-portal.georgetown.edu/). The mRNA expression and clinical data were downloaded from TCGA-LUAD (https://portal.gdc.cancer.gov/), Gene Expression Omnibus datasets (GSE72094, GSE26939 https://www.ncbi.nlm.nih.gov/geo/), and The Genotype-Tissue Expression (GTEx https://www.gtexportal.org/home). 535 ADC samples from TCGA-LUAD, 442 ones from GSE72094, and 116 ones from GSE26939 were selected for gene expression analysis and survival analysis. Meanwhile, the mRNA levels of AHNAK2 in 59 normal samples from TCGA-LUAD and 288 normal lung tissue samples from GTEx were collected for the control group. The datasets described above were extracted, annotated, and normalized by “Strawberry Perl 5.30”.

### 2.3. Survival and Prognosis

According to the median of AHNAK2 levels, the ADC patients were divided into the high- and low-expression groups. Kaplan–Meier analyses were performed to assess the role of AHNAK2 in predicting overall survival. The intersect clinical parameters in TCGA, GSE72094, and GSE26939, including age, gender and stage, were selected for survival analysis. We used univariate and multivariate Cox regression analyses to verify whether AHNAK2 could be an independent factor in predicting prognosis. *P* value < 0.05 was considered to be statistically significant.

### 2.4. Construction and Validation the Nomogram

The GES26939 was used for primary cohort and the TCGA-LUAD and GSE72094 were selected for validation cohort. We take age, gender, stage, and AHNAK2 as the factors of the prediction model and constructed a nomogram by R language (Version 3.6.1) package “rms.” The concordance index (C-index), calibration plot, time-dependent ROC, and decision curve analysis were performed to validate the nomogram via R language.

### 2.5. GO and KEGG Analyses

We screened the relation genes of AHNAK2 expression from CCLE lung adenocarcinoma cell lines (https://portals.broadinstitute.org/ccle/) and TCGA-LUAD by *t*-test. The absolute value of the Pearson correlation coefficient greater than or equal to 0.3 (∣*r* | ≥0.3) was selected as the screening criterion. Heatmaps showed the expression relationship of several representative genes with AHNAK2. With package “clusterProfiler” in R, GO and KEGG were performed to analyze the correlated genes of AHNAK2 [20]. The enriched functions and pathways were illustrated via bubble chart, bar chart, or circle chart. *P* value < 0.05 was considered to be statistically significant.

### 2.6. Cell Culture and Transfection

The ADC cell line (A549) was purchased from Procell Life Science & Technology Co., Ltd. The cells cultured in a humidified chamber containing 5% CO_2_ at 37°C with 1640 that contains 10% fetal bovine serum. The siRNA sequences targeting AHNAK2 are as follows: 5′-GTACAACCGTGTTCTTTGA -3′, 5′-GCCTAAGATTAAGCTTCCA-3′, and 5′-GTGCTCAGGTTGAAAGTCA-3′. For transfections, AHNAK2 siRNA and control siRNA were performed with Lipofectamine™ RNAiMAX (Invitrogen) according to the manufacturer's instructions. The cells were cultured continuously for 48 hours after transfection.

### 2.7. Immunofluorescence Assay

Cells were fixed with 4% paraformaldehyde in PBS for 30 min at room temperature after 48 hours' transfection and then permeabilized with 1% Triton X-100 in PBS for 15 min. Then, using 5% Normal Goat Serum (NGS) in PBS, the cells were blocked for 30 min and incubated with anti-AHNAK2 antibody (HPA004145; Sigma-Aldrich, St. Louis, MO, USA, diluted 1 : 100) overnight at 4°C. Secondary antibody incubation was performed by using Alexa Fluor 488-conjugated goat anti-rabbit antibodies (Rockland Immunochemicals Inc., Limerick, PA, U.S.A, diluted 1 : 1000) for 1.5 h at 37.0°C water bath. When each step was completed, the cells were washed 3 times with PBS, except before incubating the primary antibody. In all, the nucleus was stained by 5 mg/mL DAPI. Then, the slides were mounted and visualized using a Nikon confocal microscope. Finally, ImageJ was used to analyze the intensity of fluorescence.

### 2.8. Cell Wound Healing Assay

The A549 cells were cultured in a 6-well plate and used for wound healing assay after 24 hours' transfection. A tip of 20 *μ*L was performed to draw a straight line in each well and then washed cells with PBS. The remaining cells were cultured for 48 h in a humidified chamber containing 5% CO2 at 37°C with 1640 that containing 1% fetal bovine serum. We observed and pictured the wound healing by an optical microscope (Olympus, Japan).

### 2.9. Statistical Analyses

The SPSS 22.0 statistical program was used for IHC statistical analysis. The rest of statistical analyses were implemented in R language (version 3.6.1). Pearson's *χ*^2^ test, *t* test, or Fisher's exact test were performed to detect the significance of relationship between the variables. If it is not specified, *P* value < 0.05 was considered to be statistically significant.

## 3. Results

### 3.1. AHNAK2 Upregulated in Lung Adenocarcinoma Samples and Associated with Poor Prognosis

**Figure 1:**
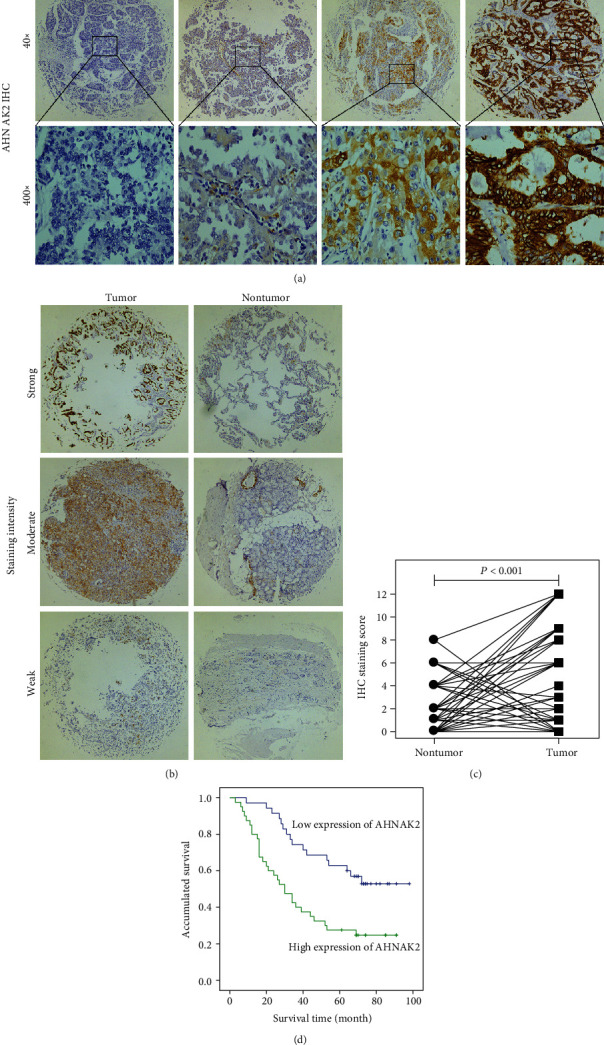
Immunohistochemical analysis of the expression of AHNAK2 in LUAD specimens. (a) Representative staining intensity images of AHNAK2 in tumor samples(40x: 40 times magnification, 400x: 400 times magnification). (b) Representative pictures of AHNAK2 with different staining intensities in tumor tissues compared with adjacent normal tissues. (c) The paired difference plot showed that AHNAK2 was highly expressed in ADC tissues (*P* < 0.05 was considered to be statistically significant). (d) Kaplan–Meier survival curve revealed that high AHNAK2 expression was significantly correlated with poor prognosis.

To investigate the correlation of AHNAK2 with prognosis in LUAD, we analyzed 80 samples from the tissue microarray. Among them, 5 cases were removed from the study cohort due to incomplete clinical data or absence of tumor tissue samples. As shown in Figure 1(a), the intensity of staining was divided into four levels. Of the 75 cases, 12 cases had no complete adjacent normal tissue specimens. The paired difference plot was performed to compare the expression of AHNAK2 in 63 tumors to that in adjacent normal tissues. The result showed that AHNAK2 was highly expressed in ADC tissues compared with the adjacent normal tissues (*P* < 0.001, Figure 1(c)). Several representative immunohistochemically stained specimens were shown in Figure 1(b). Moreover, we summarized the clinicopathological data through statistical analysis with SPSS 22.0. Kaplan–Meier survival curves revealed that high AHNAK2 expression was significantly associated with poor overall survival (*P* = 0.002, Figure 1(d)). As shown in Table 1, univariate Cox regression revealed lymph node metastasis (*P* < 0.001), TNM stage (*P* = 0.004), and AHNAK2 expression (*P* = 0.017) were significantly correlated with prognostic. Furthermore, we demonstrated that AHNAK2 (*P* = 0.005) was an independent prognostic indicator for overall survival through the multivariate Cox regression (Table 2).

**Figure 2:**
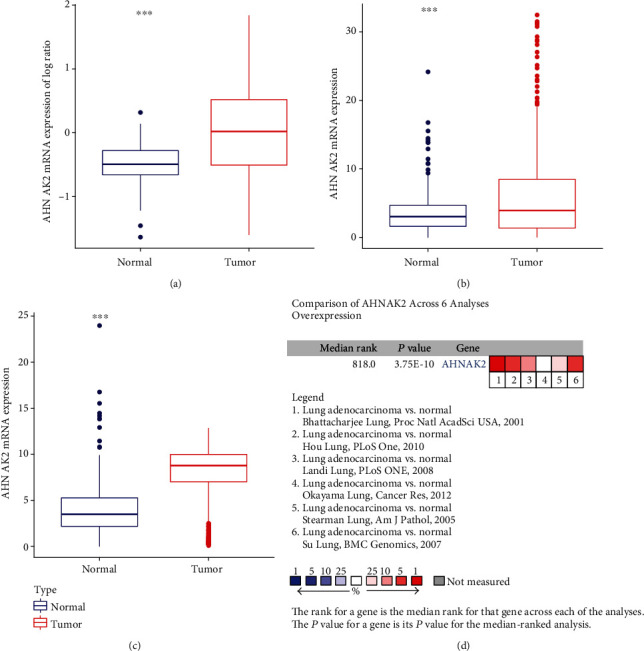
Detection of AHNAK2 protein and mRNA levels. (a) The analysis of AHNAK2 protein level in CPTAC-LUAD demonstrated that compared with normal tissues, AHNAK2 was highly expressed in tumor samples (^∗∗∗^*P* < 0.001, normal samples (*N*) = 102, tumor samples (*T*) = 109). (b) In the TCGA-LUAD and GTEx-LUNG cohorts, the mRNA levels of AHNAK2 were significantly upregulated in tumor tissues (^∗∗∗^*P* < 0.001, *N* = 347, *T* = 535). (c) In GEO (GSE72094, GSE26939) and GTEx-LUNG datasets, the mRNA expression levels of AHNAK2 were also found upregulated in ADC tissues (^∗∗∗^*P* < 0.001, *N* = 288, *T* = 558). (d) Oncomine database showed that AHNAK2 was significantly overexpressed in lung adenocarcinoma.

**Figure 3:**
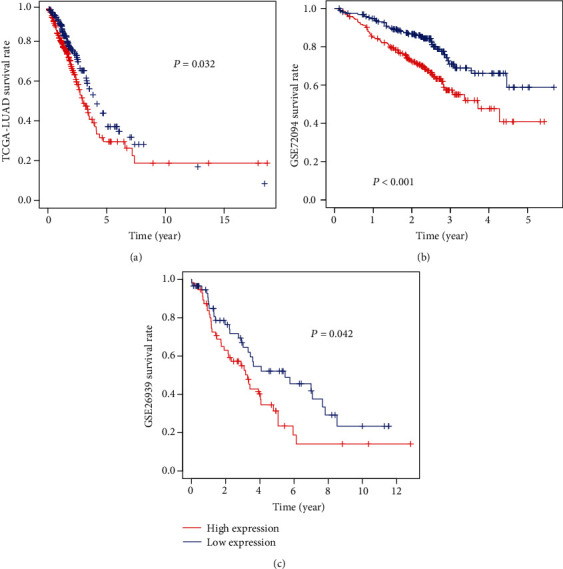
Kaplan–Meier survival curves for high AHNAK2 expression versus low AHNAK2 expression. (a) High AHNAK2 expression was significantly associated with poor overall survival in the TCGA-LUAD cohort (*P* = 0.032, samples number (*n*) = 477). (b) High AHNAK2 expression was significantly correlated with poor prognosis in the GSE72094 dataset (*P* < 0.001, *n* = 386). (c) High AHNAK2 expression was associated with poor OS in the GSE26939 cohort (*P* = 0.042, *n* = 115).

We also collected several datasets and further verified the relationship between AHNAK2 and lung adenocarcinoma. The analysis of AHNAK2 protein level in CPTAC-LUAD showed that compared with normal tissues, AHNAK2 was highly expressed in tumor samples (Figure 2(a)). Moreover, we detected the expression of mRNA in TCGA-LUAD, GEO (GSE72094, GSE 26939), and GTEx datasets; the mRNA levels of AHNAK2 were also upregulated in tumor tissues (Figures 2(b) and 2(c)). Consistent with the above results, AHNAK2 was significantly overexpressed in lung adenocarcinoma in the Oncomine database (Figure 2(d)). These observations revealed that AHNAK2 might contribute to the progression of LUAD. Furthermore, Kaplan–Meier survival curves were performed to investigate the correlation of AHNAK2 with overall survival. The missing and less-than-30-day data were removed from the TCGA-LUAD, GSE72094, GSE26939. As shown in Figure 3, high expression of AHNAK2 was apparently associated with poor prognosis (*P* < 0.05). In addition, univariate and multivariate Cox regression showed that AHNAK2 could be an independent prognostic indicator (Table 3).

### 3.2. Construction and Validation of a Predictive Nomogram

**Figure 4:**
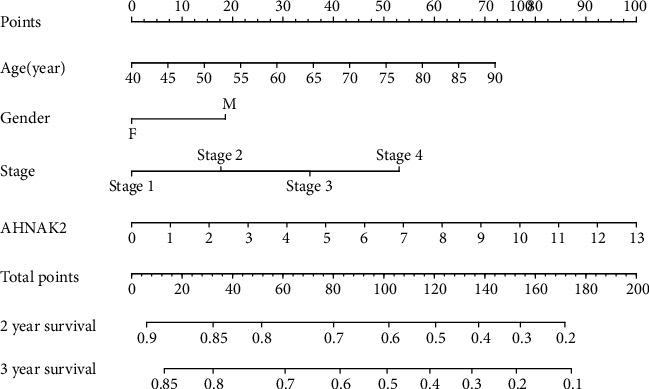
The nomogram was constructed based on four factors for predicting 2- or 3-year survival in ADC.

**Figure 5:**
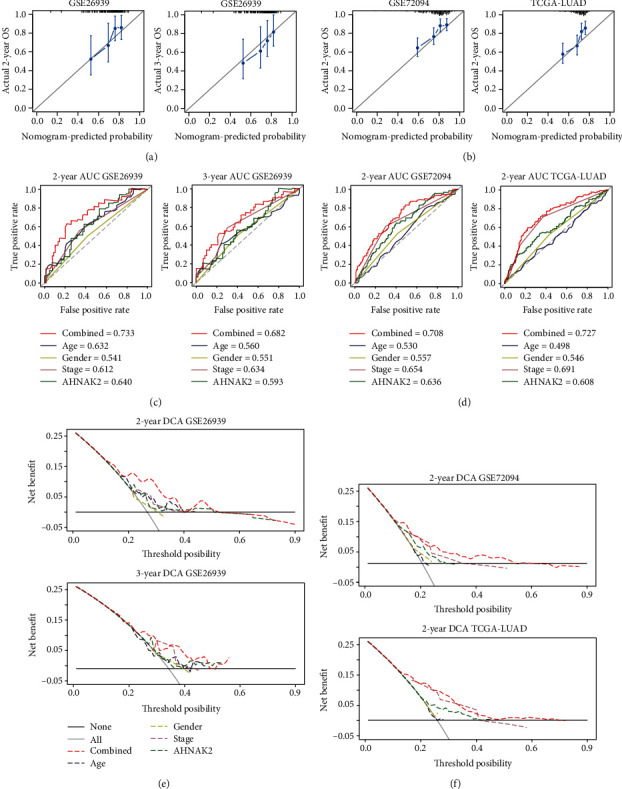
Evaluation of the value of nomogram in predicting prognosis. (a) The calibration plots of internal validation in GSE26939 showed well consistency in predicting 2- and 3- year survival. (b) The external verification of the TCGA-LUAD and GSE72094 datasets revealed an optimal agreement in 2-year survival. (c) The 2-year and 3-year AUCs of combined model in GSE2693 were 0.733 and 0.682, respectively, indicating good predictive values for survival. (d) The 2-year AUCs of a combined model in GSE72094 and TCGA-LUAD were 0.708 and 0.727, respectively, revealing a good predictive performance. (e) Decision curve analysis (DCA) of the prediction model in GSE26939 showed the best net benefit for predicting survival, especially for 2-year survival. (f). The 2-year decision curve analysis (DCA) of the prediction model in TCGA-LUAD and GSE72094.

We constructed a nomogram that included age, sex, stage, and AHNAK2 in the GSE26939 dataset (Figure 4). Next, the GSE26939 was used for internal validation; meanwhile, GSE72094 and TCGA-LUAD were selected for external verification. The C-index of the combined model was 0.663, 0.674, and 0.656 for GSE26939, GSE72094, and TCGA-LUAD, respectively. The calibration plots of GSE26939 showed a good consistency in predicting 2- and 3-year survival (Figure 5(a)). The external verification of two datasets also revealed an optimal agreement in 2-year survival (Figure 5(b)). The 2-year AUC of combined model for GSE26939, GSE72094, and TCGA-LUAD was 0.733, 0.708, and 0.727, respectively (Figures 5(c) and 5(d)). The 3-year AUC of the combined model in GSE26939 was 0.682 (Figure 5(c)). Furthermore, decision curve analysis (DCA) of the combined model showed the best net benefit for predicting survival (Figures 5(e) and 5(f)). In summary, the nomogram demonstrated that the construction with AHNAK2 could be an optimal model in predicting OS, especially for 2-year survival.

### 3.3. The Related Genes of AHNAK2 in CCLE and TCGA

**Figure 6:**
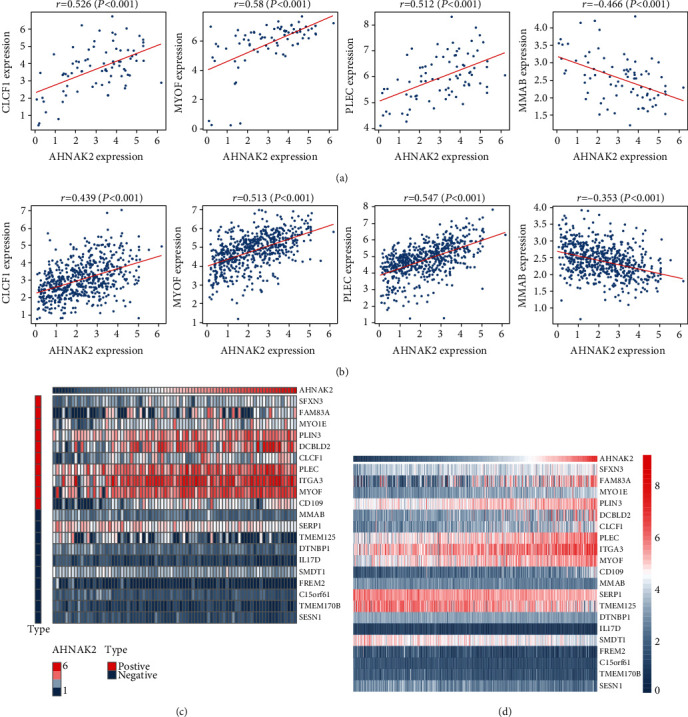
Relation genes of AHNAK2 in CCLE-LUAD cell lines and TCGA-LUAD. (a) Several related genes in CCLE-LUAD cell lines were shown in Pearson's correlation analysis chart (*r*: correlation coefficient, *P* < 0.05 was considered to be statistically significant). (b) Several related genes in TCGA-LUAD were shown in Pearson's correlation analysis chart. (c) Heatmaps showed the expression relationship of several representative genes with AHNAK2.

We screened the relation genes of AHNAK2 expression from CCLE ADC cell lines and TCGA-LUAD by *t*-test. The absolute value of the Pearson correlation coefficient greater than or equal to 0.3 (∣*r* | ≥0.3) was selected as the screening criterion. A total of 1323 genes in CCLE and 705 genes in TCGA were extracted by package “limma” of R language. Several related genes in the CCLE and TCGA datasets were shown in Pearson's correlation analysis chart (Figures 6(a) and 6(b)). Heatmaps showed the expression relationship of several representative genes with AHNAK2 (Figures 6(c) and 6(d)). Then, 148 AHNAK2-related genes of which intersection in TCGA-LUAD and CCLE-LUAD cohorts were selected for further analysis.

### 3.4. Functional Enrichment Analysis of AHNAK2

**Figure 7:**
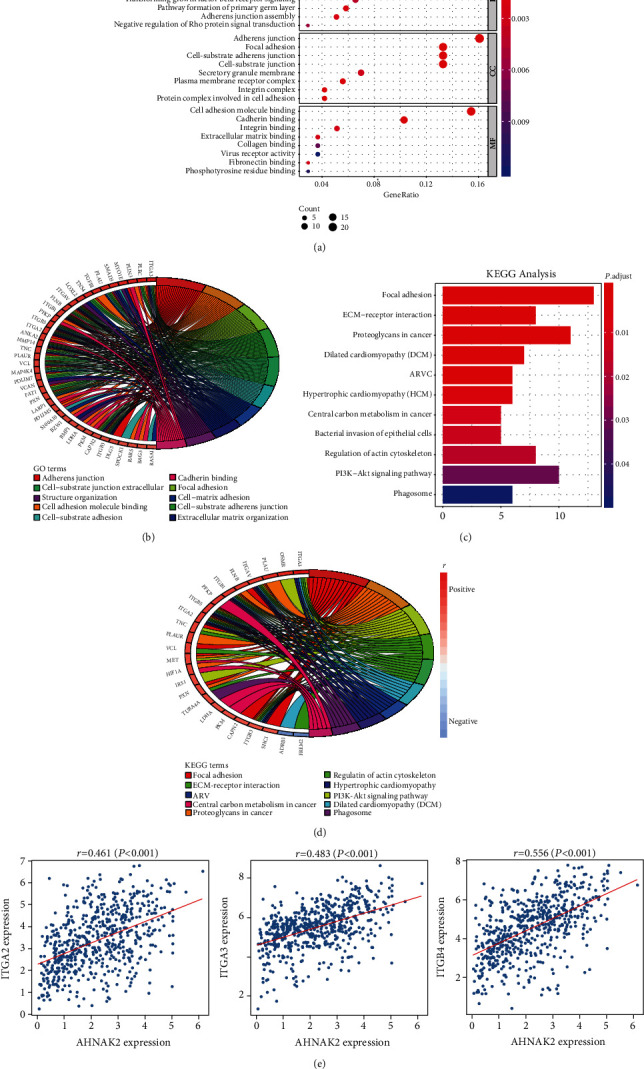
Functional enrichment analysis of AHNAK2 in ADC. (a) GO analysis of the 148 genes showed that AHNAK2 was closely correlated with adhesion function in LUAD. (b) Circle chart revealed ten GO functions of the most enrichment gene numbers, most of which were closely related to adhesion. (c) Bar chart showed the enrichment pathways of KEGG analysis. Focal adhesion, ECM-receptor interaction, and proteoglycans in cancer suggested the best optimal correlation with AHNAK2. (d) The circle chart showed ten most relevant enrichment pathways, including focal adhesion and ECM-receptor interaction. Meanwhile, the chart revealed the corresponding relationship of the gene signatures with signal pathways. (e) AHNAK2 was closely related to several integrin family molecules (ITGA2, ITGA3, and ITGB4), which play an important role in ECM-receptor interaction and focal adhesion formation (*r*: correlation coefficient, *P* < 0.05 was considered to be statistically significant).

With package “clusterProfiler” in R, GO and KEGG were performed to analyze the 148 correlated genes of AHNAK2. Biological process (BP), cellular components (CC), and molecular function (MF), as the methods of GO annotations, were performed to reveal the function of AHNAK2 in ADC. We found that AHNAK2 was closely related to adhesion structure and function in ADC, such as cell-matrix adhesion, adherens junction, focal adhesion, integrin binding, and extracellular matrix binding (Figures 7(a) and 7(b)). Moreover, the KEGG analyses revealed similar results with GO analyses. As shown in the bar chart and circle graph, AHNAK2 was significantly associated with the pathway of focal adhesion, ECM-receptor interaction, and proteoglycans in cancer (Figures 7(c) and 7(d)). In addition, through the Pearson correlation analysis, we found that the expression of AHNAK2 was significantly correlated with several integrin family molecules (ITGA2, ITGA3, ITGB4, etc.), which played an important role in ECM-receptor interaction and focal adhesion formation (Figure 7(e)). In conclusion, these results indicated that AHNAK2 might be involved in the regulation of adhesion and migration in ADC.

### 3.5. AHNAK2 Knockdown Inhibits Migration in ADC Cells

**Figure 8:**
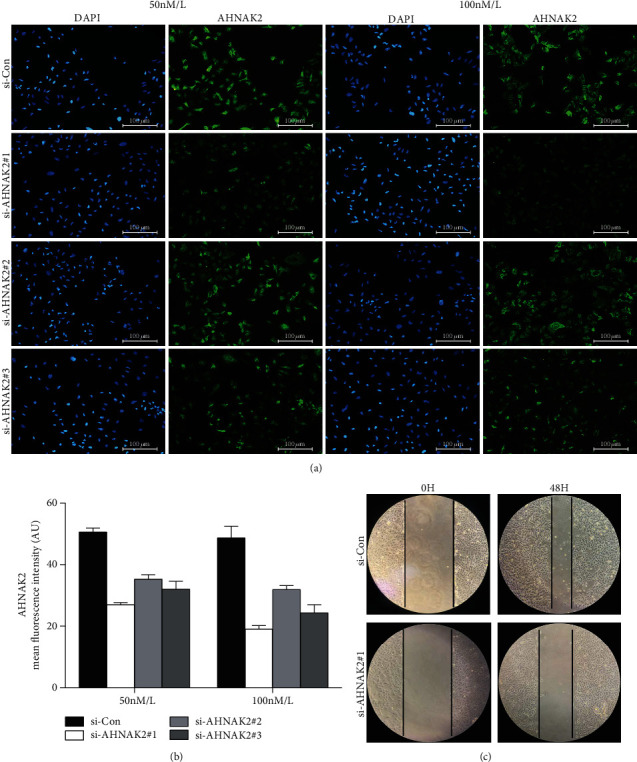
AHNAK2 knockdown inhibits migration in ADC cells. (a) Immunofluorescence was performed to reveal the effectiveness of different siRNAs. (b) Mean fluorescence intensity showed that the group of AHNAK2-siRNA#1 (100 nmol/L) had the best knockdown effect. (c) Wound healing assay showed depletion AHNAK2 inhibited A549 cells migration.

AHNAK2 siRNAs were used in order to further verify our hypothesis. A549 cells were transfected with two different concentrations (50 nmol/L, 100 nmol/L) of AHNAK2 siRNAs to knockdown endogenous AHNAK2. After 48 h, we analyzed the cellular proteins levels by immunofluorescence assay (Figure 8(a)). As shown in the histogram, we found that the levels of AHNAK2 were obviously downregulated with AHNAK2-siRNA#1(100 nmol/L). Therefore, AHNAK2-siRNA#1(100 nmol/L) was used for the next experiments (Figure 8(b)). Furthermore, wound healing assay showed depletion of AHNAK2-inhibited A549 cell migration (Figure 8(c)). To sum up, AHNAK2 might be an active regulator in cell migration of ADC cells, and the results preliminarily proved our guess.

## 4. Discussion

Lung adenocarcinoma progression is associated with alterations in assorted oncogenes and tumor suppressors [21]. Despite diagnosis and treatment of ADC being significantly improved, the prognosis of ADC is still not optimistic. Furthermore, the underlying mechanism of ADC has not been completely elucidated, which has hampered targeted therapy of ADC [22]. Therefore, the priority of ADC research is to discover the novel molecular markers associated with the prognosis of ADC. In recent years, more and more attention has been paid to the role of AHNAK family in tumor progression. Upregulation of AHNAK was significantly associated with poor prognosis of laryngeal carcinoma, mesothelioma, and pancreatic ductal carcinoma [23–25]. Interestingly, in melanoma, breast cancer, gastric cancer, and lung cancer, AHNAK acted as a suppressor in regulation of tumor progression [26–29]. However, as a homologous gene of AHNAK, the relationship between AHNAK2 and tumorigenesis was consistent in recent researches. AHNAK2 upregulated and promoted tumor progression in multiple cancers, such as melanoma, renal clear cell carcinoma, thyroid cancer, and pancreatic ductal carcinoma [13–16].

In this study, we investigated the potential role of AHNAK2 in ADC progression and detected the expression of AHNAK2. It showed that the levels of AHNAK2 were upregulated in ADC tissues. And we found that high AHNAK2 expression was obviously correlated with poor prognosis. Through univariate and multivariate Cox analyses, we demonstrated that AHNAK2 could be an independent survival prognostic factor in ADC. Moreover, with age, sex, stage, and AHNAK2, as the parameters of an integrated model, we constructed a nomogram in the GSE26939 dataset to predict 2- or 3-year OS. Calibration plots, AUC, and decision curve analysis showed that the nomogram could be an optimal model, especially for predicting 2-year survival. These results revealed that AHNAK2 was closely related to tumor progress and poor prognosis in lung adenocarcinoma. While, in future studies, more independent datasets and clinical samples should be collected to validate the role of AHNAK2 in ADC.

Recent researches reported that AHNAK2 could be intertwined with periaxin (PRX) and regulate the junction of extracellular matrix and cytoskeleton [30]. Fibroblast growth factor 1 (FGF1) is a growth factor of the nonclassical release pathway and plays an important role in regulating the MAPK-ERK signaling pathway, cell growth, tumor invasion, and angiogenesis [31]. Kirov et al. suggested that AHNAK2 and FGF1 were colocated near the cell membrane and played a key role in the regulation of FGF1 nonclassical transport [32]. A study showed that multiple sites in the CRU region of AHNAK2 could be methylated by SMYD2, thus participating in the regulation of cell adhesion, cancer cell migration, and invasion [10]. ANHAK2 was also found to promote cell migration in melanoma and renal clear cell carcinoma [13, 14]. As known, metastasis and local spread are the main reasons for the poor prognosis of lung cancer [33]. The adhesion junction between cells and extracellular matrix is the basis of tissue integrity and human health and plays a key role in cell proliferation, maintenance of activity, differentiation, and migration. Once the adhesion junction is abnormal, serious pathological changes such as tumor proliferation and metastasis will occur [34, 35].

Interestingly, through functional enrichment analysis, we found that AHNAK2 was closely related to the regulation of cell adhesion, especially to focal adhesion and extracellular matrix receptor interaction. Furthermore, through siRNA and wound healing assay, we have preliminarily verified that AHNAK2 may be an important regulator in cell migration of ADC cells. Cells contact with ECM through ECM receptors, including integrin, discoid domain receptor (DDR), collagen, and cell surface proteoglycan receptors, among which integrin is undoubtedly the most important one [36]. Through the Pearson correlation analysis, we found that AHNAK2 was significantly correlated with several integrin family molecules (ITGA2, ITGA3, ITGB4, etc), which played an important role in ECM-receptor interaction and focal adhesion formation. Recently, it has been reported that C-reactive protein (CRP) induced upregulation of ITGA2 and matrix metalloproteinase-9 (MMP-9) expressions by activating focal adhesion kinase, pilin, and ERK signal pathway, thus promoting the invasion of breast cancer cells [37]. Leng et al. reported that ITGB4 could interact with epidermal growth factor receptor (EGFR) and promote hepatocellular carcinoma lung metastases by activation of the FAK–AKT pathway [38]. These results showed that AHNAK2 might be an active regulator in cell migration. However, the molecular mechanism and signal pathway of AHNAK2 regulating cell migration need to be further studied in the future.

## 5. Conclusion

Our research revealed that AHNAK2 was upregulated in LUAD samples and related to poor prognosis. Moreover, AHNAK2 could be an independent prognosis factor for lung adenocarcinoma. In addition, we found that AHNAK2 is an important component in the regulation of cell migration. To summarize, our study showed that AHNAK2 may be a novel biomarker in LUAD and revealed the potential mechanism of AHNAK2 in LUAD progression which could provide new insights for target therapy.

## Figures and Tables

**Table 1:** Survival status and clinicopathological parameters in ADC specimens.

Characteristics	Total	Survival status		*P* value
		Died, *n* = 46	Alive, *n* = 29	
Gender				0.482
Female	37	21	16	
Male	38	25	13	
Age (years)				0.482
<60	37	21	16	
≥60	38	25	13	
Tumor size (cm)				1.000
≤3	23	14	9	
>3	52	32	20	
Lymph node metastasis				<0.001^∗^
Absent	37	15	22	
Present	38	31	7	
Distant metastases				0.078
Absent	65	37	28	
Present	10	9	1	
TNM stage				0.004^∗^
I-II	38	17	21	
III-IV	37	29	8	
AHNAK2 expression				0.017^∗^
Low	35	16	19	
High	40	30	10	

Statistical analyses were performed by the Pearson *χ*^2^ test. ^∗^*P* < 0.05 was considered to be statistically significant.

**Table 2:** Contribution of various potential prognostic factors to survival by Cox regression analysis in ADC specimens.

	Hazard ratio	95% confidence interval	*P* value
Lymph node metastasis	0.446	0.214-0.928	0.031^∗^
TNM stage	2.028	0.993-4.143	0.052
AHNAK2 expression	2.405	1.300-4.451	0.005^∗^

Statistical analyses were performed using log-rank test.

^∗^ *P* < 0.05 was considered to be statistically significant.

**Table 3:** Univariate and multivariate Cox regression showed AHNAK2 could be an independent prognostic indicator.

	TCGA-LUAD (*n* = 469)			GSE72094 (*n* = 385)			GSE26939 (*n* = 98)		
	HR	95% CI	*P* value	HR	95% CI	*P* value	HR	95% CI	*P* value
Univariate									
Age	1.006	0.990-1.023	0.449	1.497	1.028-2.181	0.035^∗^	1.023	0.997-1.049	0.085
Gender	1.032	0.752-1.416	0.845	1.009	0.989-1.029	0.389	1.67	0.982-2.838	0.058
Stage	1.693	1.464-1.957	<0.001^∗^	1.653	1.383-1.976	<0.001^∗^	1.317	0.984-1.764	0.064
AHNAK2	1.038	1.022-1.054	<0.001^∗^	1.251	1.083-1.445	0.002^∗^	1.147	1.034-1.273	0.009^∗^

Multivariate									
Age	1.008	0.993-1.024	0.292	1.614	1.097-2.375	0.015^∗^	1.024	0.996-1.053	0.093
Gender	0.93	0.674-1.282	0.656	1.004	0.984-1.024	0.716	1.358	0.779-2.367	0.281
Stage	1.695	1.462-1.966	<0.001^∗^	1.694	1.408-2.038	<0.001^∗^	1.337	0.977-1.83	0.069
AHNAK2	1.038	1.020-1.056	<0.001^∗^	1.207	1.050-1.387	0.008^∗^	1.135	1.028-1.253	0.012^∗^

^∗^ *P* value < 0.05 was considered to be statistically significant.

## Data Availability

The data used to support the findings of this study are included within the article.
